# Current Status of PRP Manufacturing Requirements & European Regulatory Frameworks: Practical Tools for the Appropriate Implementation of PRP Therapies in Musculoskeletal Regenerative Medicine

**DOI:** 10.3390/bioengineering10030292

**Published:** 2023-02-24

**Authors:** Patrick Sebbagh, Alessandro Cannone, Gerald Gremion, Vincent Gremeaux, Wassim Raffoul, Nathalie Hirt-Burri, Murielle Michetti, Philippe Abdel-Sayed, Alexis Laurent, Nathalie Wardé, Lee Ann Applegate

**Affiliations:** 1 Regenerative Therapy Unit, Plastic, Reconstructive & Hand Surgery Service, Lausanne University Hospital, University of Lausanne, CH-1066 Epalinges, Switzerland; 2 NAD Consulting Sàrl, CH-1208 Geneva, Switzerland; 3 Sport Medicine Unit, Division of Physical Medicine and Rehabilitation, Swiss Olympic Medical Center, Lausanne University Hospital, University of Lausanne, CH-1011 Lausanne, Switzerland; 4 Lausanne Burn Center, Lausanne University Hospital, University of Lausanne, CH-1011 Lausanne, Switzerland; 5 DLL Bioengineering, STI School of Engineering, Ecole Polytechnique Fédérale de Lausanne, CH-1015 Lausanne, Switzerland; 6 Manufacturing Department, LAM Biotechnologies SA, CH-1066 Epalinges, Switzerland; 7 Center for Applied Biotechnology and Molecular Medicine, University of Zurich, CH-8057 Zurich, Switzerland

**Keywords:** biologicals, clinical cytotherapies, good manufacturing practices, musculoskeletal affections, orthobiologics, platelet-rich plasma, process standardization, regenerative medicine, regulatory frameworks, therapeutic products

## Abstract

Providing accurate and up-to-date practical tools enabling oversight of platelet-rich plasma (PRP) legislation and of the appropriate standards to be implemented for its manufacture and use in Europe is a demanding task. This is due to rapid medico-technological advancements, slowness and disparity in legislation updates and enforcement between member states, and many reported gray-zone practices, notably for autologous PRP use. The levels of risk associated with blood manipulation processes generally dictate the manufacturing requirements for PRP preparations, which have gradually shifted toward good manufacturing practices (GMP) for standardization and overall quality enhancement. This work firstly outlines Western European and Swiss legislation for PRP products/preparations, providing key simplified information and recommendations for medical doctors seeking to implement this biological-based therapy for safe use in hospital settings, clinics, or private offices. This work secondly shows the importance of PRP-based product manufacturing standardization, which subsequently enables sound clinical evaluation of therapeutic interventions. Although the applicable legal bases provide guidelines for GMP manufacturing infrastructure and basic process design, paramount importance is set on the definition of workflows, technical specifications, and key parameters for PRP preparation and delivery. Overall, the development of simple and robust technologies and processes for PRP preparation is critical for guaranteeing the high therapeutic quality of the intervention, in collaboration with qualified GMP manufacturing platforms. Importantly, this work aims to serve as a practical tool for clinicians based in Western Europe who are willing to appropriately (i.e., administratively and technically) implement autologous PRP treatments in musculoskeletal regenerative medicine workflows, to ensure they make informed and optimal regulatory or process-based decisions.

## 1. Introduction

Regenerative medicine consists of the replacement or help in the regeneration of cells, tissues, or organs in the human body to establish or restore normal function, thus presenting tremendous curative and preventive potential [1]. It includes cell therapy, gene therapy, tissue engineering, biomedical engineering techniques, and treatments involving biological products, which encompass platelet-rich plasma (PRP) [2,3,4]. Although such techniques may not all be categorized as curative, many may provide strong ancillary therapeutic support and may significantly improve the quality of life of treated patients [2]. In common affections, such as articular lesions/inflammation or tissue scaring, regenerative medicine options may reduce the need for traditional medication administration and may provide a holistic solution in complex pathophysiological situations [5,6]. With highly versatile therapeutic approaches, which have been constantly improving for several decades, regenerative medicine can potentially address all systems and tissues of the human body [7,8,9,10,11].

Clinical results yielded by the application of cell-based therapies have been evolving rapidly since the 1980s, particularly in burn patient care and in orthopedics [2,6,9,12]. The use of biological products/cells has demonstrably improved clinical outcomes, such as those reported for PRP administration in burn patients and for cutaneous wound management [8,13]. PRP alone or in combination with rapidly isolated skin keratinocytes from severe burn patients have notably demonstrated that simple biological-based therapeutic approaches could procure significant advances in severe burn victim care [13]. As the time to initial treatment is a critical factor for these patients, the rapid use of such simple preparations has shown significantly increased healing rates and pain reduction, which represents major milestones [13]. From the technical standpoint, a recent study has shown that preparations based on rapidly isolated patient keratinocytes could be standardized and implemented for manufacture in a good manufacturing practice (GMP) facility, guaranteeing appropriate levels of process and environmental control [14].

The therapeutic use of PRP and similar orthobiologics for managing a variety of musculoskeletal conditions is rapidly increasing worldwide and was drastically enhanced by the availability of CE-marked or approved kits for ease of preparation of blood products [15,16,17,18,19,20,21]. This was made possible notably due to the absence of FDA regulations regarding the manufacture of the final orthobiologic product, while the focus was historically set on the used device [16,17,22]. Importantly, this lack of recommendations and guidelines potentially introduces unbearable iatrogenic risks (e.g., viral transmission), which are exacerbated for the use of PRP in non-medical settings [23]. Within appropriate clinical settings, the autologous local use of PRP has been shown to be safe, minimally invasive, and effective to promote tissue relief from inflammation and pain [8]. Notably, hundreds of thousands of athletes and patients are treated annually with PRP, with more than 80,000 athletes treated in the USA alone already a decade ago [24]. Interestingly, this treatment remains allowed by the World Anti-Doping Agency (WADA), while the use of growth factors remains prohibited [24,25].

Professional organizations have promoted the use of PRP, such as the American Academy of Orthopaedic Surgeons, which has included PRP in the 2021 practice guidelines for osteoarthritis (OA), or the American Medical Society for Sports Medicine, which supports advancements in the domain of clinical orthobiologics. In addition, the American Academy of Orthopaedic Surgeons has published a consensus paper aiming to improve and accelerate the clinical evaluation, clinical use, and multifactorial optimization of biological-based therapies for musculoskeletal diseases and affections [26]. From a therapeutic standpoint, PRP applications have frequently been used within tissues with low intrinsic healing potential and for indications such as tendinopathies, calcaneal and plantar fasciitis in the foot, muscle strains, ligament sprains, articular cartilage injuries and degeneration, or local preparation before hair transplants [7,27,28,29,30,31,32,33,34,35,36].

Accurately providing an appropriate and current oversight of PRP legislation in Europe is complex due to the rapidly evolving laws and regulations and the high number of individual member states or jurisdictions [3,34,37]. The same complexity applies to the manufacturing standards for PRP and for its clinical use in regenerative medicine [34]. Multiple factors are interconnected and notably comprise the rapid medico-technological advancements, the slowness and disparity in legislation updates, and the inhomogeneous enforcement of the laws between member states. Additionally, many borderline or gray-zone practices have been reported in the field for autologous PRP injections [37]. From a technical standpoint, safety- and quality-oriented risk analyses for blood manipulation are used to determine the appropriate manufacturing process requirements for PRP-based products/preparations. In recent European regulations, these process requirements have gradually shifted toward good manufacturing practices (GMPs) for standardization purposes and with the objective of obtaining overall quality enhancements for the finished PRP products/preparations [37,38,39,40,41,42,43,44,45,46,47,48].

The background to the present study consists of the documented high heterogeneity in the application of legal and regulatory requirements for PRP treatment implementation in Western Europe [24,34,37,38]. A tangible need was thus identified to establish a summarized source of clear information on the legal and regulatory bases and key stakeholders, which should be preliminarily consulted by medical professionals willing to implement PRP treatments in regenerative medicine workflows. In parallel, high heterogeneity has been documented on the technical side for autologous PRP obtention and use, where the application of various and non-standardized methods and protocols results in high variability in the PRP products that are clinically administered [4,15,16,18,19,20]. Based on this background and on the extensive translational experience available in Lausanne for the GMP manufacture and clinical use of autologous PRP, process-based and quality-oriented considerations were set forth as an evidence-based source for the same professionals interested in the new implementation of PRP treatments in their practice [13,35].

This study firstly aims to outline and concisely summarize the key points of the current European and Swiss legislation for PRP products/preparations [39,40,41,42,43,44,45,46,47]. From a general technical standpoint, if the blood is manipulated in a closed system, most often in aseptic conditions using equipment that is a medical device (MD), some requirements (e.g., authorization from local authorities) might be not applicable/necessary for PRP preparation activities [39,40,41,42,43,44]. This is, however, not the case in Switzerland, where an authorization from Swissmedic (i.e., the national health authority for therapeutic products) is mandatory to manufacture blood products [35,46,47]. Importantly, most European countries consider PRP as a “non-standardized medicinal product”, for which GMP standards must be applied [34,45]. Specifically, Directive 2005/62/EC clearly states that good practice guidelines (GPG) based on the principles of GMP must be implemented, and the guidelines clearly define the standards, which are very close to GMP [40,41]. Switzerland has adapted this same approach in the Therapeutic Products Act (TPA) and requires similar practices and standards [46,47]. This study secondly shows the importance of PRP-based product preparation (i.e., manufacturing process and controls) standardization, which is a prerequisite for sound clinical evaluation of high-quality therapeutic interventions [9]. Despite detailed descriptions of the necessary GMP manufacturing infrastructure in the applicable framework documents, few elements pertaining to process design are provided [39,40,41,49]. Therefore, the choice of the exact manufacturing process and protocol is the responsibility of the manufacturer, which should carefully consider and optimize the workflows, technical specifications, and key parameters of PRP preparation and clinical delivery [9,15,16,35].

Importantly, an appropriate equilibrium must be found between the maintenance of the optimal biological properties of a given preparation and the safety-oriented requirements of the related manufacturing workflow, as well as feasibility in clinical routine [9,35]. Specifically, despite important regulatory and manufacturing constraints, the central aspect of successful PRP-based therapeutic approaches relies on the clinically-oriented and scientifically optimized product development process [49,50]. The continuous development of simple, yet robust technologies and processes for autologous PRP manufacture represents a key driver for delivering high therapeutic quality and cost-effective interventions in the clinic [51,52]. This may notably be performed in collaboration with qualified GMP manufacturing contractors [15,35]. Indeed, recent evolutions in the applicable legal bases make it highly advisable to standardize PRP preparation protocols under GMPs, making it possible to adapt end-products to different clinical settings [3,37,49]. Most importantly, qualified personnel are required for the administration of PRP and biologicals to assure proper safety of the clinical intervention and to avoid iatrogenesis [3,12,17].

The major contributions and novelty of the present study consists of the availability (i.e., in a single source) of up-to-date legal and regulatory elements to be firstly considered by European clinicians in order to appropriately introduce PRP treatments in their practice from an administrative and legal standpoint. Secondly, a comparative discussion about the possibilities and limits existing in various Western European countries for autologous PRP preparation should provide important insights to the readers regarding the technical/quality aspects of product processing. In this context, the present work sets forth the significant advantages that may be procured by the use of GMP-accredited platforms for autologous PRP manufacture (i.e., process validation and standardization, insurance of traceability and consistency). Overall, the present study should significantly contribute to ensure that new physicians lay down the appropriate administrative and technical bases for PRP implementation in musculoskeletal regenerative medicine, which are prerequisites to providing safe and effective therapeutic interventions to their patients.

## 2. Aims, Scope, and Methodology of the Study

The first specific aim of the present study was to perform an updated analysis of the legal and regulatory frameworks that are currently applicable in Western European countries (i.e., Switzerland, Germany, France, Italy, and Spain) for autologous PRP manufacture and clinical human use. The objective of this part of the study was to provide a summary of the official sources and a currently valid catalogue of the applicable legal, regulatory, and guidance documents to physicians considering the implementation of autologous PRP treatments in their practice. From a methodological point-of-view, the compiled legal base documents were prospectively gathered from the official national websites of the Western European countries of interest and that of the European Union. This ensured that the considered documents were up-to-date and currently in force. These sources were cross-checked with the publicly available online records and registries of the national health authorities of the countries of interest.

The second aim of the present study was to perform an updated analysis of the responsible parties (i.e., health authorities, regulators, license or accreditation providers) in the same Western European countries. The objective of this part of the work was to provide a summary of the official sources and a currently valid catalogue of the parties who should be contacted by individual physicians interested in the clinical implementation of autologous PRP protocols. This aspect was considered as especially important for the readers due to the inhomogeneity of the requirements and practices across the analyzed jurisdictions. From a methodological point-of-view, the responsible parties were identified by a prospective analysis of the publicly available online records and registries of the national health authorities of the countries of interest.

Simplified and illustrated workflows are presented in Figures S1 and S2 for an optimal description of the general design of the study and presentation of the protocol used in Lausanne for the GMP preparation of autologous PRP. Overall, the present study and article were designed as practical tools for physicians based in Western Europe. Specifically, the present work was meant to ensure that the readers integrate the following notions and key messages:

(i) Basic scientific knowledge on the nature, growth factor contents, and known therapeutic effects of autologous PRP preparations in regenerative medicine;
(ii) Awareness of the currently applicable legal bases and regulatory frameworks in their respective jurisdictions;
(iii) Awareness of the currently competent authorities and entities that should be contacted in case of doubts on the requirements to fulfil before implementing autologous PRP treatments in their practice.

## 3. Biological-Based Therapeutics for Enhanced Patient Management: Overview of the Known PRP Attributes and Actions

**Figure 1:**
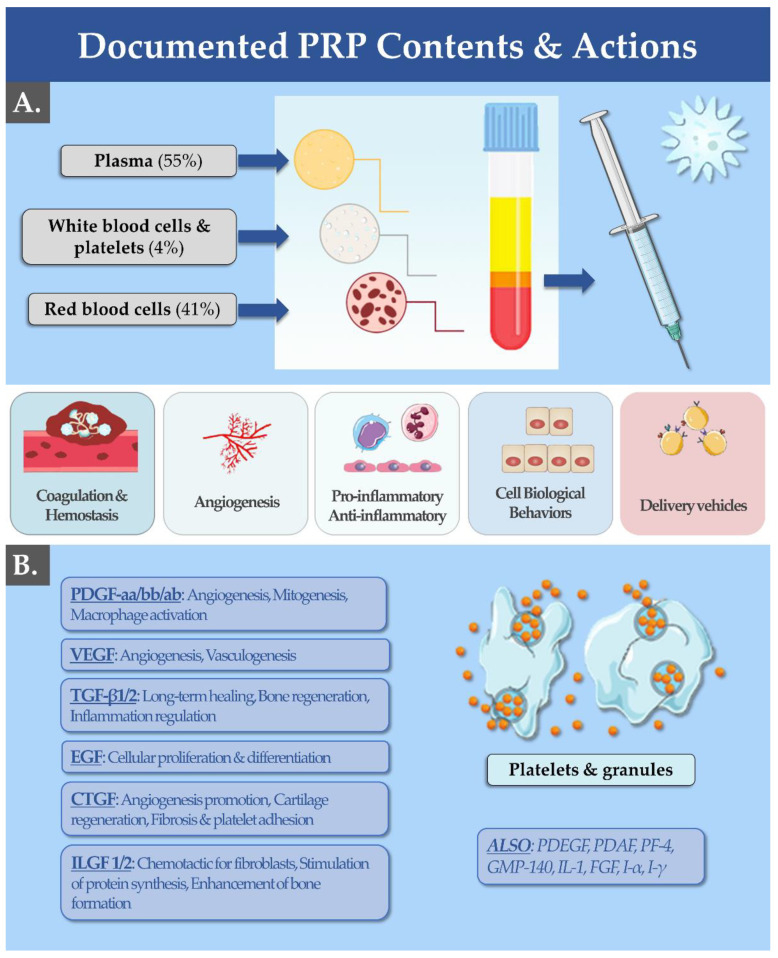
(**A**) PRP is isolated from a whole blood sample taken by venous access (i.e., ≥20 cc needed depending on the indication). Concentrated platelets are obtained by centrifugation of autologous blood in a 2-step procedure, aiming to eliminate RBC and to prepare a 2–5X concentrate of platelets in the isolated plasma volume. The mechanisms of action of PRP in regenerative medicine are implicated at all stages of wound healing (e.g., coagulation, angiogenesis, inflammation) due to the various growth factors available in the biological preparations. (**B**) Listing of the most common growth factors found in PRP, along with their documented effects. Cc, cubic centimeters; CTGF, connective tissue growth factor; EGF, epidermal growth factor; FGF, fibroblast growth factor; GMP-140, platelet alpha-granule membrane protein; IL, interleukin; ILGF, insulin-like growth factor; PDAF, platelet-derived angiogenesis factor; PDGF, platelet-derived growth factor; PF, platelet factor; PRP, platelet-rich plasma; RBC, red blood cells; TGF, transforming growth factor; VEGF, vascular endothelial growth factor.

Physiologically, platelets possess several functions, which include aggregation during primary haemostasis, release of inflammatory mediators, and stimulation of tissue healing [16,53,54]. Such effects are taken into account for the classification of platelet-based therapeutic preparations for human use (e.g., autologous PRP) as medicinal products [4,17,34]. In vivo, platelets become activated upon detection of the slightest damage to the endothelium [16,54]. In response to an injury, platelets release chemokines, growth factors, angiogenesis stimulators and inhibitors, bactericidal/fungicidal/virucidal proteins, immune mediators, coagulants, anticoagulants, and fibrinolytic proteins [55,56,57] (Figure 1).

Platelets then express adhesion factors in order to adhere to the injured endothelium (Figure 1B). In the context of regenerative medicine, the platelet growth factors are of particular interest [54,57,58]. Platelets notably synthesize epidermal growth factor (EGF), hepatocyte growth factor (HGF), insulin-like growth factor, and transforming growth factor (TGF) (Figure 1B) [54,57]. These growth factors promote cell proliferation, cell migration, and collagen synthesis (Figure 1B). In addition, platelets contain vascular endothelial growth factor (VEGF), fibroblast growth factor (FGF), platelet-derived growth factor (PDGF), angiostatin, and endostatin, which help to improve the perfusion of damaged tissues and therefore contribute to rapid regeneration (Figure 1) [4,24]. These angiogenic factors allow the development of blood microcirculation and increase capillary permeability [24]. Both types of described factors (i.e., growth factors and angiogenic factors) are contained in the granules of platelets (Figure 1B) [57]. In PRP preparations, the method of extraction of these active agents consists of the centrifugation of whole blood to separate the plasma from the cells and to allow their release, making them freely available in the finished product form (Figure 1A) [50].

## 4. Updated Analysis of the Applicable European & Swiss Legal Frameworks and Requirements for the Manufacture of Clinical Grade PRP

As PRP constitutes a blood-derived product and is classified as a medicinal product, its use is strictly regulated in the European Union (EU) [34]. These stringent requirements were meant to ensure proper control of all the aspects related to preparations, storage, and distribution, and thereby guaranteeing the quality and safety of the finished PRP products [39,40,41,42]. In particular, the applicable regulatory framework concerning the quality and safety aspects surrounding blood is Directive 2002/98/EC (Table S1) [39]. Furthermore, Directive 2005/62/EC and its amending Directive 2016/1214 set out the rules in regards to the quality system standards and specifications for blood establishments (Table S2) [40,41]. This encompasses a broad range of necessary practices and controls, including technical requirements for the collection of blood and testing, license requirements, qualified personnel and responsible persons, quality system management, traceability of the products, and the guidelines to ensure that all of the steps are accomplished safely (Tables S1 and S2) [39,40,41]. The directives are acknowledged in the various EU member states with specific internal regulations [38]. This inhomogeneity in the EU regulatory ecosystem may lead to the community legislators to intervene rapidly in order to even out the “rules of engagement” of this particular class of biologicals [34,37].

In Switzerland, blood-derived products, including PRP, are considered as non-standardized medicines, therefore they are generally subject to authorization according to the law on therapeutic products [46,47]. The aim of the applicable documentation is to set forth the regulatory framework applicable to products obtained following the manipulation of whole blood (e.g., PRP) using means of separation such as medical devices [47]. However, the regulations concerning the accreditation and commercialization of the devices (i.e., as pertains to European Regulation 2017/746) are not specified [43,44,46,47]. Importantly, the medical devices used for PRP preparation often belong to various risk categories due to the heterogeneity of manufacture, assembly, and operating instructions, and therefore require specific and non-uniform regulatory pathways for obtaining the CE mark [44,59]. The scope of Directive 2002/98/EC is to ensure that blood and its components are of comparable quality and safety throughout the blood transfusion chain in all member states, bearing in mind the freedom of movement of citizens within the community territory (Table S1) [39].

In the Summary Report following the Meeting of the Competent Authorities on Blood and Blood Components concerning PRP (i.e., 11–12 October 2012, Brussels) the commission communicated that the considered procedure should be considered within the scope of the directives on blood [39,40,41,42]. Notwithstanding, the competent national authorities have subsequently provided the opinion that it is practically difficult to bring this procedure fully in line with what was set forth by the 2002 Directive [34,39]. Therefore, it was decided that this procedure would constitute the object of ad hoc regulations by the EU in the future [37]. Some authorities have put more stringent authorization measures in place, resulting in divergent levels of safety and effectiveness for patients across member states [37,38]. However, the Directive 2002/98/EC states that “*The nature of autologous transfusion necessitates a specific consideration in respect of how and when to apply the different provisions of this Directive*”, leaving some room for interpretation by hematological centers [39]. The margins for interpretation are partially filled by Directive 2005/62/EC and its amending Directive 2016/1214, which prepared rules with regard to quality system standards and specifications for blood establishments, defining additional details for the requirements already covered in Directive 2002/98/EC [39,40,41]. In particular, Directive 2005/62/EC states that good practice guidelines (GPG) shall be developed by the commission for ensuring alignment with the applicable standards and specifications [40]. Those guidelines are to be based on the principles of good manufacturing practices (GMP) [40].

Good manufacturing practices are a set of quality rules to be applied in the production and distribution of pharmaceutical products [49]. GMPs are used in Europe and in Switzerland and are similar or very similar in content to good manufacturing practices in the USA [35,49]. GPGs (and GMPs) are strict recommendations, whereas current good manufacturing practices (cGMPs) are fully-fledged legal texts [49]. GMPs define production, testing, and quality assurance objectives to ensure that pharmaceutical products are safe for consumption. The regulation uses the terms “pure, safe, and effective”. GMPs set out rules to ensure that the basic principles detailed in Table S2 are met [49].

## 5. Updated Analysis of the Competent Health Authorities and Regulatory Structures/Entities for PRP in Switzerland and in Europe

In Switzerland, Swissmedic or the Swiss Agency for Therapeutic Products is responsible for monitoring the market for therapeutic products [49]. It was founded in 2002 following the merger of the Intercantonal Office for Drug Control (IOMC) and the Therapeutic Agents Unit of the Federal Office of Public Health (FOPH). Since its creation in 2002, Swissmedic has been responsible for the implementation of the law on therapeutic products [46].

In Europe, in addition to the national agencies that are able to authorize the marketing of a pharmaceutical product in their territory, there is a central agency, the European Medicines Agency (EMEA) [60]. This agency is an umbrella structure that allows parallel European registrations. Obtaining a marketing authorization issued by the European Medicines Agency allows the product to be marketed in all European countries [60].

### 5.1. Health Authorities in Switzerland with Competence over PRP

Switzerland is currently not an EU member state, yet similar Swiss regulations concerning blood-derived products exist, as compared to the principles expressed in the above-mentioned directives (Tables S1 and S2) [39,40,41,42,43,44,45,46,47]. In Switzerland, the law on therapeutic products (i.e., LPTh or TPA, RS 812.21) constitutes the legal basis for chemical and biotechnological drugs, medical devices, vaccines, implants, diagnostics, and blood products, for which the preparation process must follow the standards set forth for the preparation of medicinal drugs [46]. PRP is considered as a “non-standardized” medicinal product since it is not industrially produced, and it is a patient-specific preparation [50]. Indeed, patient-specific therapeutic preparations can seldom be standardized in a manner comparable to normal medicinal products, since their origin usually incurs high biological variability [49]. PRP preparations therefore cannot be defined as formula-related medicinal products. Although manufactured following a physician’s prescription for his/her specific patients, PRP preparations are not usually produced according to a classical and standardized process [35]. They are usually not manufactured in public pharmacies or hospital pharmacy departments, which is the case for formula-based preparations [46].

However, since PRP preparations have historically been considered to be medicinal products, they are currently subjected to authorization following Art. 9, para. 1 of the Therapeutic Products Act (TPA, RS 812.21) [46]. This is, however, not the case if the considered preparations fall under the exemption ruling stated in Art. 9, para. 2 TPA. Specifically, this exemption is applicable to formula-related medicinal products, preparations to be used in clinical trials, and non-standardized medicinal products [46]. The Therapeutic Products Act was revised in 2019 and the classification possibilities for non-standardized medicinal products were diversified. Consequently, some patient-specific preparations can currently be classified as non-standardized medicinal products following Art. 9, para. 2, let. E TPA, and such is the case for PRP [46]. Based on the fact that non-standardized medicinal products are distinct from formula-based preparations, the manufacture and distribution activities require a Swissmedic establishment license. Furthermore, such preparations are to be manufactured following the GMP requirements listed in Annex 1 of the Ordinance on Licensing in the Medicinal Products Sector (MPLO; RS 812.212.1) [47].

Specifically, in order to protect the intended recipients, mandatory authorizations are now required for certain non-standardized medicinal products, including PRP [47]. However, the Therapeutic Products Act describes some settings in which non-standardized medicinal products may conditionally continue to be manufactured and used without regard to the new provisions (Table 1) [46]. These exceptions encompass association with particular equipment and environments, patient management during blood processing, assuring product sterility and rapid use, and overall patient safety.

In scenarios not covered by the new provisions, the person performing the treatment remains subject to the basic and general medical duty of care and is responsible for guaranteeing the safety, quality, and efficacy of the administered treatment [46,47]. There are activities that may be allowed only with proper authorization and these conditions are related to timing of the preparation and the product administration [47]. Specifically, these provisions apply when the sterility of the product could be a factor of concern and when the preparations are not destined to the same patient, as presented more specifically in Table 2 below.

As stated for Switzerland, the national regulatory body that oversees the domain of PRP manufacture and use is Swissmedic. In accordance with Article 58 of the Swiss Law on Therapeutic Products, Swissmedic is responsible for hemovigilance [46]. Therefore, all institutions that are duly authorized to handle blood and blood products (e.g., blood transfusion services) must implement a reporting procedure and must employ a responsible person for hemovigilance, who appropriately reports adverse incidents related to production and distribution [46,47]. The Swiss approach is therefore similar to the one adopted in Germany, notably with the implementation of GMP standards and accreditations/authorizations issued by regulatory authorities.

### 5.2. Health Authorities in Germany with Competence over PRP

In Germany, the law governing blood components is the Decree of 20 December 2007 (i.e., number 261) associated with the Health Ministry Decree of 3 March 2005, which constitutes the key document regulating the preparation and use of blood components. The law classifies blood components as medicinal products and an authorization is therefore necessary for marketing, according to the German Medicinal Products Act (i.e., AMG, Arzneimittelgesetz). Consequently, GMP and good distribution practices (GDP) apply to the manufacturing/manipulation and distribution phases.

The national organization that issues the required authorizations is the Paul Ehrlich Institute (PEI) within the German ministry of health. In Germany, transfusion of blood components is specifically regulated by the Transfusion Act (i.e., TFG Transfusionsgesetz). Within this legal framework, section 18 of the TFG must be considered with great care, as the hemotherapy guidelines are particularly important.

### 5.3. Health Authorities in France with Competence over PRP

In France, implementation of the “Blood System” is the responsibility of the Direction Générale de la Santé (DGS). Hemovigilance and the related systems are the responsibility of the ANSM (Agence nationale de sécurité du médicament et des produits de santé/National Agency for Drug Security and Health Products). Practically, the French blood system relies on a network of over 800 blood transfusion centers. Transfusion control procedures and systems mainly cover the “Produits Sanguins Labiles” (PSL, labile blood products), which are obtained from donors and are intended to be transfused to a patient for therapeutic purposes (article L1221–8 of the Code de la Santé Publique (Code of Public Health), amended by the law 2011–2012 of 29 December 2011, Article 5). These processes usually involve whole blood, plasma, and human blood cells. The corresponding list and PSL attributes are specified by the ANSM, which consults the Etablissement français du sang (EFS, French Blood Establishment). The EFS is a French public institution purposed with an array of advisory functions and activities, notably those related to the therapeutic use of human blood. Accordingly, whereas stable preparations for medical blood transfusions are considered as drugs, PSLs (i.e., including all blood components for topical application) are not regulated in a similar manner and are instead assessed and authorized case-by-case.

### 5.4. Health Authorities in Italy with Competence over PRP

Italian legislation for “blood components for non-infusion/transfusion” (e.g., topical preparations) use encompasses all the components used in all therapeutic scenarios in which blood components are not transfused into the patient. Instead, the blood components must be used directly in the pathologic area and may be administered locally by various means (e.g., intra-articular/deep tissue injection in orthopedics, cutaneous use in dermatology, subcutaneous use in plastic surgery, etc.). The preparation of platelet concentrates or platelet gels is not considered to fall under the category of blood derivative production due to simple processing by physically fractionating the blood into blood components. Once authorized by the competent/specific authorities and even in an outpatient setting, it is technically possible for any orthopedic surgeon to prepare concentrated platelets by using appropriate machines. Such machines normally possess a CE-marked closed circuit and make it possible to centrifuge and/or further filter the autologous blood, separating the plasma portion (i.e., portion rich in platelets) for therapeutic use.

In Italy, the notion of “minimum manipulation” is applicable to the processing of blood components that are not combined with other drugs and that are prepared rapidly. A major risk, in the case of autologous donors, would be the dysfunction of the preparation apparatus or system. Additionally, in the case where the blood components are to be used in a topical therapeutic indication, this would deviate from the classic infusion/transfusion administration route. In order to abide by the rules of minimal manipulation, concentrated platelets are to be prepared by simple physical means (e.g., centrifugation, separation), and must not be used for experimental purposes or for somatic cell therapy.

Of note, the Committee for Advanced Therapies (CAT) has identified (i.e., on 13 November 2009) “fresh and freeze-dried thrombocytes isolated from autologous or allogeneic blood” as “products intended for wound healing in orthopedic and dental surgery”. Consequently, such products were not considered as advanced therapy medicinal products at that time. Currently, the blood system regulatory framework is specified by the decree of 21 October 2005 (i.e., no. 219). While this law and the decree of 20 December 2007 (i.e., no. 261) generally refer to blood systems, the decree of 3 March 2005, specifically defines the applicable rules for blood component preparation.

In Italy, the manipulation of blood is, by law (i.e., no. 219/2005), restricted to transfusion services. The transfusion services are public institutions subject to accreditation and are usually located in hospitals. Such services are competent for all processes encompassing the collection, storage, handling, and distribution of blood and blood components. Nevertheless, it has been legally recognized that structures supplied by the transfusion services may be authorized for the “remote” outpatient preparation of blood components for topical autologous use if ad hoc agreements are established and specify the following:

(a) The transfusion service must keep full control over the remote processing of blood components, including all traceability aspects, meaning registration of the procedure;

(b) The medical professionals must provide their patients with a rapid and effective service, safe products, and tangible logistical advantages (i.e., no need for pre-donation, use of a fresh product, use of a non-frozen product, reduced need for blood harvesting).

Therefore, agreements between medical doctors and transfusion services may allow the physician to accomplish the outpatient preparation of blood components for topical use, provided that appropriate oversight is ensured by the competent transfusion service. Despite the Italian regulation being relatively lenient, all manipulation of blood components performed without the appropriate authorization is constitutive of a criminal offense. The unauthorized ambulatory use of PRP preparations without an agreement with a transfusion center is never legal, which can make this practice more heterogeneous and complex than in other European countries.

### 5.5. Health Authorities in Spain with Competence over PRP

Spain has implemented the European Directive 2002/98/EC in 2005, further specifying the technical requirements for the donation, processing, and use of whole blood and blood components. As regards the PRP manufacturing process, the platelet separation step must be performed in a closed system or in a circuit or must at least be performed in sterile/aseptic systems. Notably, all the procedures governing blood manipulation for products that are intended for transfusion are placed under the authority of blood transfusion centers (i.e., Center de Transfusión Sanguínea). However, for special procedures (e.g., autologous, intraoperative use, with small amounts for specific protocols of treatment), the Comité Cientifico para la Seguritad Transfusional (CCST, Committee for Transfusion Security) has declared that regulations for hemotherapy practices (i.e., CCST opinion in response to a request for clarification of 10 May 2004) would not apply.

In May of 2013, the Spanish Agency of Medicines and Medical Devices (AEMPS) together with different experts in the field of PRP set forth a resolution. This resolution established the classification of non-replacement therapeutic use of autologous plasma and associated fractions, components, or derivatives as a medicinal product for human use to meet specialized needs. Therein, PRP was classified as a non-industrial biological medicine intended for human clinical application. Similarly, the AEMPS has stated that in Spain, PRP therapy could not be classified as an ATMP. Thereby and with this new framework, the AEMPS aimed to ensure the quality, efficacy, traceability, information, and pharmacovigilance for PRP preparations in a similar manner to classical medicinal products. PRP preparations must therefore be considered as medicines, which incurs strict requirements for manufacturing, traceability, efficacy and safety, and patient information, implicating GMP and GDP application.

## 6. Importance of Manufacturing Process Standardization for Therapeutic Biologicals, Such as Autologous PRP

Overall, there seems to be a consensus to standardize therapeutic biological preparation manufacture, in addition to basic monitoring of the environment and infrastructure used in their preparation [16,26]. Based both on past in-house experience in the Lausanne University Hospital and on the literature, key parameters and recommendations are provided hereafter for the optimization of GMP preparation processes of PRP (i.e., open processes under grade A working environments, Figure S2, Table 3) [15,16,35,50].

When working with biologicals destined for medical treatments, the defined parameters prepared specifically based on the needs of the prescribing clinician are very important [9]. Autologous PRP products are complex and naturally variable in their composition and the need for thorough quality controls in view of clinical applications makes it crucial to demonstrate consistency in the manufacturing process [4,15,51,52]. General protocols for PRP preparation consist of blood collection and a first centrifugation step to separate the red blood cells (RBC), which is then followed by a subsequent second centrifugation step to concentrate the platelets and other components and to provide activation of the biological product (i.e., mechanical action of injection, Figure S2) [61,62,63,64,65,66].

The main parameters that may be optimized comprise the type of medical devices chosen for blood draw, variation of the relative centrifugal force, as well as temperature and time brackets for platelet isolation (Table 3) [16,59,62,66]. Even though commercially marketed PRP kits offer the possibility of rapid obtention of ready-to-use PRP suspensions, which should be sterile, several disadvantages have been identified [16,59]. Indeed, such kits often come with a high purchasing cost, limited blood draw possibilities, and variable guidelines for centrifugation steps, which are related to large variability in the final platelet concentration/purity of the PRP delivered to the patient [16]. Therefore, a variety of technical aspects make it difficult for the clinician to practically decide which commercial system would be most adequate for the considered clinical application.

## 7. Discussion

### 7.1. Current Inhomogeneity in Practices for PRP Preparation and Clinical Use in Europe

The indications for PRP use are varied and are found in the fields of plastic surgery, hand surgery, septic conditions and sports medicine, anaesthesiology, radiology, and for many specific indications such as epicondylitis, patellar tendinopathy, Achilles tendinopathy, osteoarthritis, burns, wounds, grafts, scars, and hair loss [17,67,68,69,70,71,72,73]. Therefore, guidelines to aid the safe and correct use of PRP will potentially benefit many healthcare providers within diverse environments, whether in hospitals, clinics, or private practices [3,12,16,26,33]. Specifically, such guidelines may help to assure that the proper treatment protocol is provided for the patient and that PRP preparation is performed within registered and licensed laboratories [16,37].

Careful consideration of the applicable European and national regulations regarding the manufacture and use of PRP enable us to draw several conclusions on the use of such blood products (Tables S1 and S2) [39,40,41,42]. In general, the operational principle is that, based on the type of blood component to be used, authorizations may be required from the national authorities responsible for transfusion activities or for therapeutic products [39,46,47]. In Italy, exceptions are not allowed, and blood components prepared for topical use (i.e., considered as blood products) must always be placed under the responsibility of an accredited blood transfusion service. This oversight structure is always necessary, regardless of the type of product, the amount of blood and blood-derived product, and the retained processing protocol for clinical use [37].

In Europe, and depending on the country, specific cases or settings make it possible for blood components to be considered either as blood products or, alternatively, as medicines [37]. Therein, and depending on the quantity of retrieved blood, the manufacturing process and the applied clinical protocol, PRP preparations may be used by the physician under his/her responsibility in a less restrictive manner (Table 1) [47]. Therefore, the medical professional should submit the considered protocol to the competent regulatory authority in order to receive advice on the appropriate use of specific blood components. Specifically, the physician should determine if an authorization is required or if the considered form of therapy may be classified as an act to be performed freely under his/her control and responsibility.

The reported high inhomogeneity in the EU legislative ecosystem regarding the management of products obtained from whole blood processing will probably lead the legislators to soon work toward harmonization of the “rules of engagement” of this particular class of biologicals/cell therapy [34,37]. Indeed, PRP technologies are being promoted to European medical doctors based on the technical aspects of separation devices that have a CE mark [16,59]. However, the different EU member states are not regulating the use of such devices in a homogenous manner. For instance, the Italian framework allows the physical and contractual delegation of product manufacture and administration to physicians, yet the responsibility remains that of the blood transfusion service. In other European states, ambulatory use under the direct responsibility of the physician is possible. Therein, high variability exists between the procedures, which depend on the protocol used by the physician, the specific therapeutic application, the amount of blood, the manufacturing system, etc. [37].

Therefore, throughout European countries, there is a general rule based on the same directive, yet in each country, the “legal” way to use PRP depends on the retained manufacturing and clinical protocols [37,38,39]. Because of this, the regulation tends to be heterogeneous and quite complex to correctly assimilate by individual healthcare providers. These factors sometimes lead to requirements for specific authorizations or the need for other clinicians or structures (i.e., transfusion services) to be involved in the PRP processing steps. Furthermore, these requirements make it somewhat difficult for clinicians in certain countries to use autologous PRP within their operative and clinical practices [37,38]. In this sense, it would certainly be helpful for all the countries that accept a CE mark for a device to subsequently function similarly with respect to processing specifications/standards, simplifying, and therefore favoring the use of PRP [59]. This concerted approach bares the potential of harmonizing the practices in clinical settings, providing tangible advantages for patient care [16].

### 7.2. PRP Preparation and Use in Switzerland: Toward Harmonization and Standardization under GMPs

In Switzerland, the Therapeutic Products Act includes possible scenarios in which, under certain conditions, non-standardized medicinal products may continue to be manufactured and used by physicians without regard to the new provisions (Table 1) [46,47]. In scenarios not covered by the new provisions, the person performing the PRP treatment remains responsible for guaranteeing the safety, quality, and efficacy of the administered treatment. Focusing specifically on the Swiss legislation, it can be concluded that Switzerland is interpreting and implementing elements of Directive 2002/98/EC following a similar approach to the one introduced in Germany, which considers blood components as medicinal products, therefore requiring GMP standards [39,46].

### 7.3. PRP Manufacturing Devices and Ready-to-Use Kits: Technical Focus of Regulators

From a technical standpoint, the currently marketed medical devices for PRP preparation claim to have different concentration possibilities for platelets, yet it is not clinically confirmed that a higher concentration of platelets correlates with enhanced efficacy [16,59,74,75]. Indeed, the literature covering clinical work suggests that the main consensus is to have a 2–3-fold platelet concentration factor and that the technique used to produce the final PRP product should be gentle enough to avoid any damage to the biological constituents [16,50,76]. In practice, platelet concentration factors in PRP can vary from 2–12 times and some studies have even reported a 0.52 value from the baseline, even though evoking standardization methods [15,16,17]. It is not clear from available clinical studies that higher platelet doses or concentrations provide an increased clinical benefit [27,75].

Based on the commercial availability of over 40 systems for the preparation of PRP, all claiming safe production and the potential to promote healing, there seems to be a necessity for specific regulation and oversight of their use [16,59]. Indeed, despite its simple concept and rapid manufacturing technique, the use of PRP injections is invasive and bears a certain level of risk [24,36]. Medically speaking, there are several contraindications for PRP use, which include hypofibrinogenemia, anticoagulation, hemodynamic instability, sepsis, infection, chronic liver problems, platelet dysfunction and critical thrombocytopenia, and the use of corticosteroids or nonsteroidal anti-inflammatory drugs, for example [36]. In addition, if PRP is used for facial rejuvenation, there could be a risk of a viral flare-up due to tissue manipulation and inflammatory reactions [15,17,36]. Therefore, the therapeutic use of PRP should be delegated to medically-associated facilities, whether private practices, private clinics, or hospitals, and not within basic esthetic centers, which can be seen in countries with lenient regulation (e.g., USA) [22,23].

Since PRP final products themselves are not regulated, it is only the medical devices, such as the centrifuge and the preparation kits, which are regulated and these do not require clinical data for registration [16,59]. Specifically, this is possible due to the fact that these systems are intended for autologous use with minimal sample manipulation and are considered as a low-risk biological product [43,44]. Therefore, PRP and the individual components of platelets and plasma are often exempt from quality control measures in practice [51,52]. Although the use of these systems is not regulated to date, there are several recent events which may change the relevant regulatory ecosystem in the future, as unobstructed use may have severe consequences on patient safety [37]. A notable example of iatrogenesis cause by the inappropriate use of PRP has been documented in an esthetic spa, with the transmission of HIV in New Mexico [23]. There are also significant implications from the financial side of healthcare systems, as these PRP preparations can be very expensive (i.e., 500–2500 US dollars per treatment) and are mainly not covered by insurance [22,59].

### 7.4. Transitioning toward GMP-Accredited Platforms for PRP Preparation: Enhanced Traceability and Quality of Orthobiological Care

For the provision of the highest available quality of autologous PRP, it is important that the laboratory tasked with PRP preparation operates under the requirements of GMP for small quantities of medicines [35]. The main burdens lie in the ability to show that the laboratory premises and staff meet GMP requirements, that the entity has a quality assurance system, and that the centrifuge settings, temperature, and equipment remain unchanged so that the product specifications are also standardized [49]. For private clinics or private practices wishing to produce their own PRP, it is always possible to acquire equipment that has been validated for this purpose [59]. Indeed, the centrifuge and preparation kits should be simple to use with as little handling as possible, be a “closed” system (i.e., the sample is never exposed to air in order to avoid any contamination of the PRP that will be injected into the patient), be sterile, and have the medical CE mark as a guarantee of safety [43,59].

Overall, the competent supervisory authorities are entitled to request the user (i.e., physician and/or manufacturer) to submit data and evidence concerning the quality, safety, and efficacy of the preparation under consideration [37,38]. In addition, the competent authorities may prohibit the manufacture of these preparations and the practice of PRP injections if these conditions are not found to be met [47]. From a technical point-of-view, high attention should be paid to the standardization of PRP-based product preparation methods and final product attributes, which enables the sound clinical evaluation of treatment efficacy [16]. Despite the relatively elevated fixed costs of PRP manufacturing under GMPs as compared to the use of kits, the increasing regulatory pressure is orienting healthcare providers toward the augmentation of quality standards [16,37,49]. Therein, the appropriate and widespread use of standardized GMP processing workflows in musculoskeletal regenerative medicine applications bears the potential of lowering healthcare costs by systematically enhancing the therapeutic quality of orthobiologic clinical interventions [50].

### 7.5. Comparative Aspects of PRP Manufacture under GMPs in Lausanne or Using PRP Kits and Closed Systems

A main technical difference between the standardized protocol adopted in Lausanne for the GMP preparation of PRP and the use of alternative systems consists in the fact that the GMP process is not “closed” (Figure S2) [50]. Indeed, several steps of open-container manipulation and liquid handling are performed, which increase the product contamination (i.e., particulate and/or microbiological) risk level as compared to the use of a “closed” system (i.e., where the blood products are always contained during processing). However, the contamination risks are brought down to negligible levels in the validated GMP process, as all open-container operations are carried out in a class A environment under a laminar flow (Figure S2) [49]. Specifically, this enables more flexibility and ease of processing of the fluids as compared to closed systems, as the manipulator retains better direct control over the process [50].

Several reports and studies have described high rates of clinical success for PRP treatment in a number of indications, yet it is now well-known that both the preparation method and the clinical administration modalities play critical roles in the efficacy of the intervention [77,78,79,80]. Therefore, many calls for the standardization of PRP preparation methods have been documented in order to globally enhance the related clinical success rates [16]. Within this objective, the use of a standardized GMP manufacturing workflow for autologous PRP (i.e., as described for the Lausanne University Hospital) presents multifactorial advantages and technical superiority, as detailed hereafter (Figure S2). Specifically, best-in-class reproducibility may be obtained by the use of an accredited GMP platform, which is inherently qualified and validated to perform the manufacturing process [49]. This is due to the fact that the equipment is appropriately designed and maintained for the needs of the operations, and that such operations are carried out by trained and qualified personnel [9]. This aspect is drastically different from the use of PRP kits by individual physicians at the bedside of patients, where numerous variability factors may come into play and affect the attributes of the final product [16,59].

Furthermore, maximal traceability and safety can be demonstrably achieved with GMP production of PRP due to the documentary requirements for manufacturing and testing activities [9]. Therefore, the use of a standardized GMP process leads to the generation of exhaustive batch records that contain valuable data for further process optimization or for efficacy-related studies [9]. Furthermore, the control requirements of GMP processes enable us to set forth a higher level of safety of the finished products as compared to a PRP kit. Therein, in-process and post-process sampling and testing of the materials enable us to document and demonstrate the safety and quality of the administered preparations [49,50]. Overall, the proposed solution for the GMP preparation of autologous PRP is in line with current reflections and recommendations regarding the standardization of processes in the field of cell-based regenerative medicine [35,49,50].

### 7.6. Study Significance and Identified Limitations

Overall, this study was meant to significantly contribute to the existing professional body of knowledge around autologous PRP, notably to ensure that new physicians dispose of simple tools to proceed with the appropriate administrative and technical bases prior to PRP implementation. Such preliminary elements are considered to be of the utmost importance for the provision of legal, safe, and effective therapeutic interventions. Importantly, this study also aimed to set forth several process-based and quality-oriented considerations for the same medical professionals with the demonstration and the discussion of the various advantages of using a GMP platform instead of CE-marked PRP kits for product preparation.

The main identified limitations of this study are related to the highly dynamic nature of the legal and regulatory ecosystems for autologous PRP in Europe [37]. Namely, it is probable that several elements referenced in the present study will be outdated in a matter of years or even months due to iterative updates in the respective laws, frameworks, and guidelines.

## 8. Conclusions and Perspectives

The present study has strongly emphasized the current disparities (i.e., legal, administrative, technical) that exist in the field of autologous PRP use for human clinical practices in Western Europe. Specifically, the diversity in the applicable legal and regulatory frameworks was described for Switzerland, Germany, France, Italy, and Spain, along with the diversity in the documented medical practices that may be encountered in each country. Importantly, it was discussed that the harmonization of practices or the use of appropriate infrastructure and quality systems are essential in the domain of autologous PRP for the assurance of optimal safety and efficacy of the orthobiologic interventions. Overall, appropriate and systematic consideration of the available technical, regulatory, and clinical hindsight available for autologous PRP use needs to be integrated by clinicians willing to implement such treatments. These elements are considered to be central in view of maintaining and enhancing the quality level of the available personalized regenerative medicine healthcare. This objective may be further attained tangibly through the holistic consideration of specific clinical cases, the effective dialogue between attending physicians and PRP manufacturers, and the use of standardized autologous PRP preparation workflows.

## Figures and Tables

**Table 1:** Exceptions to new rulings on the authorization requirements for PRP manufacture in Switzerland, in the case where the criteria are respected (e.g., the physician must ensure the safety, quality, and efficacy of the treatment). PRP, platelet-rich plasma.

Exception ID	Description of the Exceptions
1.	The preparation of autologous products fully complies with the equipment manufacturers’ instructions.
2.	The patient does not leave the room where the initial blood material is withdrawn until final administration, including during the preparation.
3.	The blood is prepared in the same room under the direct supervision of the patient’s physician.
4.	The preparation is performed in a closed system if the product must be sterile.
5.	The administration to the patient happens immediately after the preparation is performed and under the supervision of the patient’s physician or personnel under her/his direct responsibility following her/his detailed instructions.
6.	The product cannot be used for the treatment of other patients.
7.	The product cannot be distributed to the patients.
8.	The procedure and the relevant documentation must be in writing and part of the quality system.

**Table 2:** PRP manufacturing activities allowed only with an authorization from Swissmedic (i.e., the physician must ensure the safety, quality, and efficacy of the treatment). PRP, platelet-rich plasma.

Activity ID	Description of the Activities
1.	Preparation and/or administration after a period of storage or not immediately.
2.	Additional manipulation of the obtained fractions out of the closed system.
3.	Addition of active ingredients or substances that are not part of the production process, according to the instructions from the equipment vendor.
4.	Preparation of multiple doses at any stage of the manipulation.
5.	Allogenic preparation (i.e., preparation destined to a patient different than the donor).

**Table 3:** Key manufacturing and control parameters influencing PRP quality, as well as recommendations and possibilities for PRP quality optimization. PPP, platelet-poor plasma; PRP, platelet-rich plasma; RBC, red blood cell.

Parameter	Definition of Critical Process Items, Parameters, and Quality Optimization Options
Blood Draw Devices and Anticoagulants	Anticoagulant vacutainer tubes and blood collection needles of the appropriate size and medical device grade quality. Anticoagulant choice for the preservation of platelet function, integrity, and morphology (e.g., citrate and sodium citrate dextrose are recommended).
Blood Draw Homogenization	Blood collections in tubes or bags should be thoroughly homogenized by gentle inversion at least 5–10 times, depending on the size and the volume, for mixing of the anticoagulant throughout. This needs to be assured to avoid any small fibrin clot formation, which would influence the total platelet count.
Blood Centrifugation-Step N°1	Validation of the equipment/method for the initial centrifugation to assure optimal separation of platelets from the whole blood. A platelet count in the RBCs and within the supernatant would allow this step. Altering the centrifugation time and rotor speed would be indicated if the parameters have not yet been optimized. In addition, the temperature within the centrifuge should be standardized.
Blood Centrifugation-Step N°2	The second centrifugation step should be optimized based on the determination of the optimal platelet counts in PPP and PRP. A higher concentration of platelets in the upper layer (PPP) would indicate that the process parameters are not optimal.
Controls of PPP and PRP	PRP should be separated from PPP rapidly following the secondary centrifugation step, as concentrated platelets diffuse into the PPP over time and reduce the PRP preparation yield. The concentrated final PRP preparation should be appropriately re-suspended to allow homogenization before the platelet counting procedure.

## Data Availability

Data are available upon reasonable request made in writing to the corresponding author.

## Supplementary Materials

The following supporting information can be downloaded at: https://www.mdpi.com/article/10.3390/bioengineering10030292/s1, Table S1: Scope of Directive 2002/98/EC: Blood Component Quality and Safety; Table S2: Scope of Directive 2005/62/EC: Good Practice Guidelines (GPG) based on Good Manufacturing Practices (GMP); Figure S1: Schematic illustration of the design of the study; Figure S2: Schematic illustration of the PRP manufacturing process performed under GMP in the Lausanne University Hospital.

## Abbreviations

AEMPS	Spanish Agency of Medicines and Medical Devices
AMG	Arzneimittelgesetz, German medicinal products act
ANSM	French National Agency for Drug Security and Health Products
ATMP	advanced therapy medicinal product
CAT	Committee for Advanced Therapies
cc	cubic centimeter
CCST	Spanish Committee for transfusion security
CE	EU medical device conformity evaluation symbol
cGMP	current good manufacturing practices
CTGF	connective tissue growth factor
DGS	French Direction Générale de la Santé
EC	European Commission
EFS	French Blood Establishment
EGF	epidermal growth factor
EMEA	European medicines agency
EU	European Union
FDA	US Food and Drug Administration
FGF	fibroblast growth factor
FOPH	federal office of public health
GDP	good distribution practices
GMP	good manufacturing practices
GMP-140	platelet alpha-granule membrane protein
GPG	good practice guidelines
HIV	human immunodeficiency virus
IL	interleukin
ILGF	insulin-like growth factor
IOMC	Swiss intercantonal office for drug control
LPTh	Swiss law on therapeutic products
MD	medical device
MPLO	Swiss ordinance on licensing in the medicinal products sector
OA	osteoarthritis
PDAF	platelet-derived angiogenesis factor
PDGF	platelet-derived growth factor
PEI	Paul Ehrlich Institute
PF	platelet factor
PPP	platelet-poor plasma
PRP	platelet-rich plasma
PSL	labile blood products
RBC	red blood cells
TFG	Transfusionsgesetz, German transfusion act
TGF	transforming growth factor
TPA	therapeutic products act
USA	United States of America
VEGF	vascular endothelial growth factor
WADA	World Anti Doping Agency
