# Supplementary material for: Current Status of PRP Manufacturing Requirements & European Regulatory Frameworks: Practical Tools for the Appropriate Implementation of PRP Therapies in Musculoskeletal Regenerative Medicine
Source: Bioengineering (Basel). 2023 Feb 24;10(3):292. doi: 10.3390/bioengineering10030292 (PMC10044789; doi:10.3390/bioengineering10030292)
Supplement: Supplementary file 1 [file bioengineering-10-00292-s001.zip › Supplementary Materials.pdf]

Supplementary Tables

Table S1. Scope of Directive 2002/98/EC: Blood Component Quality and Safety. These regulatory framework elements are of specific importance for the appropriate setup and operation of manufacture and control activities for clinical grade PRP products/preparations. EC, European Commission; GMP, good manufacturing practices; PRP, platelet-rich plasma.

Parameter or Nomenclature Term	Nomenclature Term Definition and Specification of Directive Scope
**Technical Requirements**	Collection and testing of blood and blood components, including starting materials for medicinal products.
**License Requirements**	Insurance that appropriate mechanisms for designating, authorizing, accrediting, or licensing hematological centers exist. Insurance that the Health Authority inspections for the activities of blood establishments are performed within the scope of the Directive.
**Head Transfusion Service Designation**	Responsible for ensuring that each unit of blood or blood product is collected, controlled, processed, preserved, distributed, and assigned in compliance with the Directive.
**Qualified Personnel**	Appropriately qualified personnel involved in the collection, testing, processing, storage, and distribution of blood and blood components is necessary. Relevant training should be periodically updated and recorded.
**Quality System**	Quality systems involve all activities that determine the quality policy objectives and responsibilities and are implemented by such means as quality planning, quality control, quality assurance, and quality improvement within the quality system, taking into account the principles of good manufacturing practice (GMP) as well as the EC conformity assessment system.
**Traceability System**	Traceability of whole blood and blood components should be enforced through accurate donor, patient, and laboratory identification procedures, through record maintenance, appropriate identification, and labelling systems.<br>1. Autologous blood and blood components must be clearly identified as such and stored, transported, and distributed separately from allogeneic blood and blood components.<br>2. Autologous blood and blood components must be labelled as required by Directive 2002/98/EC and in addition the label must include the identification of the donor and the warning '*FOR AUTOLOGOUS TRANSFUSION ONLY*', ensuring that they will not be used for transfusion to other patients.
**Surveillance Procedures**	Information collection and evaluation on adverse or unexpected events or reactions resulting from the collection of blood or blood components must be performed, in order to prevent similar or equivalent events or reactions. Common systems of notification of serious adverse events and reactions linked to the collection, processing, testing, storage, and distribution of blood and blood components must be established.
**Counselling**	Insurance about when and if abnormal findings have to be reported to a particular donor.

Table S2. Scope of Directive 2005/62/EC: Good Practice Guidelines (GPG) based on Good Manufacturing Practices (GMP). These regulatory framework elements are of central importance for the appropriate manufacture and control activities for clinical grade PRP products/preparations. CAPA, corrective and preventive actions; CE, conformity evaluation; EC, European Commission; GMP, good manufacturing practices; GPG, good practice guidelines; PRP, platelet-rich plasma; QA, quality assurance.

Parameter or Nomenclature Term	Nomenclature Term Definition and Specification of Directive Scope
**Quality**	Responsibility of all employees involved in the process. Management is responsible for ensuring a systematic approach, implementation, and maintenance of a Quality System.
**Critical Process**	Instructions which are carried out in accordance with predefined standards and specifications.
**Management Review**	Performed at regular intervals to verify the effectiveness of the Quality System and to identify potential improvements, if needed.
**Independent Quality Assurance**	Support by a Quality Assurance (QA) function. Involved in all of the quality-related matters and responsible for approval of all of the appropriate quality-related documents. Independent from the processing management.
**Validation Process**	Procedures, premises, and equipment that have an influence on the quality and safety of blood and blood components need to be validated prior to introduction and be re-validated at regular intervals, determined as a result of these activities.
**Personnel**	Available in sufficient numbers to carry out the activities related to the collection, testing, processing, storage, and distribution of blood and blood components and be trained and assessed to be competent to perform their tasks. All personnel shall have up-to-date job descriptions which clearly set out their tasks and responsibilities. All personnel shall receive initial and continued training appropriate to their specific tasks. Training records shall be maintained. Training programs shall be in place and shall include good practice with content periodically assessed, and the competence of the personnel evaluated regularly. There shall be written safety and hygiene instructions in place and adapted to the activities to be carried out.
**Premises**	Premises, including mobile sites, shall be adapted and maintained to suit the activities. They shall enable the work to proceed in a logical sequence so as to minimize the risk of errors and shall allow for effective cleaning and maintenance in order to minimize the risk of contamination. Blood collection shall be carried out in an area intended for the safe withdrawal of blood from donors, appropriately equipped for the initial treatment of donors experiencing adverse reactions or injuries from events associated with blood donation and organized in such a way as to ensure the safety of both donors and personnel, as well as to avoid errors in the collection procedure. Dedicated laboratory areas exist for testing that are separate from blood donor and blood component processing areas, with access restricted to authorized personnel. Storage areas shall provide for properly secure and segregated storage including quarantined and released materials and units of blood or blood components collected under special criteria (e.g., autologous donation). Provisions shall be in place in the event of equipment or power failure in the main storage facility. An area shall be designated for safe disposal of waste, for disposable items used during the collection, testing, and processing, and for rejected blood or blood components.
**Equipment & Materials**	Equipment shall be validated, calibrated, and maintained. Operating instructions shall be available and appropriate records kept. Equipment shall be selected to minimize any hazard to donors, personnel, or blood components. Only reagents and materials from approved suppliers that meet the documented requirements and specifications shall be used. Critical materials shall be released by a person qualified to perform this task. Where relevant, materials, reagents, and equipment shall meet the requirements of European Regulation 2017/745 for medical

	devices and European Regulation 2017/746 for in vitro diagnostic medical devices or comply with equivalent standards in the case of collection in third countries. Inventory records shall be retained for a period acceptable to and agreed upon with the competent authority.
**Computerized Systems**	Software, hardware, and backup procedures must be checked regularly to ensure reliability, be validated before use, and be maintained in a validated state. Hardware and software shall be protected against unauthorized use or unauthorized changes. Back-up procedures shall prevent the loss of or damage to data at expected and unexpected down times or function failures.
**Documentation**	Documents setting out specifications, procedures, and records covering each activity performed by the blood establishment shall be in place and kept up-to-date. Records shall be legible and may be handwritten, transferred to another medium and documented in a computerized system. All significant changes to documents shall be acted upon promptly and shall be reviewed, dated, and signed by a person authorized to perform this task.
**Donor**	Procedures for safe donor identification, suitability interview, and eligibility assessment shall be implemented and maintained. The donor interview shall be conducted in such a way as to ensure confidentiality. The donor suitability records and the final assessment shall be signed by a qualified health professional.
**Traceability**	Blood collection procedures shall be designed to ensure that the identity of the donor is verified and securely recorded and that the link between the donor and the blood, blood components, and blood samples is clearly established. There shall be a system in place to ensure that each donation can be linked to the collection and processing system into which it was collected and/or processed. The sterile blood bag systems/tubes used for the collection of blood and blood components and their processing shall be CE-marked or comply with equivalent standards if the blood and blood components are collected in third countries. The batch number of the blood bag/tube shall be traceable for each blood component.
**Blood Collection Procedures**	Procedures shall minimize the risk of microbial contamination. Laboratory samples shall be taken at the time of donation and appropriately stored prior to testing/use. The procedure used for the labelling of records, blood bags/tubes, and laboratory samples with donation numbers shall be designed to avoid any risk of identification error and mix-up. After blood collection, the blood bags/tubes shall be handled in a way that maintains the quality of the blood and at a storage and transport temperature appropriate to further processing requirements and patient application.
**Laboratory Testing**	Laboratory testing procedures shall be validated before use. Each donation shall be tested in conformity with the requirements laid down.
**Validation Devices**	Equipment and technical devices shall be used in accordance with validated procedures, including measures to avoid the risk of contamination and microbial growth in the prepared blood components.
**Labelling**	At all stages, all containers shall be labelled with relevant information of their identity. In the absence of a validated computerized system for status control, the labelling shall clearly distinguish released from non-released units of blood and blood components. The labelling system for the collected blood, intermediate, and finished blood components and samples must unmistakably identify the type of content and comply with the labelling and traceability requirements. For autologous blood and blood components, the label also shall comply with Article 7 of Directive 2004/33/EC and the additional requirements for autologous donations specified in Annex IV to that Directive.

**Release**	There shall be a safe and secure system to prevent each single blood and blood component from being released until all mandatory requirements are fulfilled. Each blood establishment shall be able to demonstrate that each blood or blood component has been formally released by an authorized person. Records shall demonstrate that before a blood component is released, all current declaration forms, relevant medical records and test results meet all acceptance criteria. Before release, blood and blood components shall be kept administratively and physically segregated from released blood and blood components. In the absence of a validated computerized system for status control, the label of a unit of blood or blood component shall identify the release status.
**Storage & Distribution**	The quality system of the blood establishment shall ensure that, for blood and blood components intended for the manufacture of medicinal products, the storage and distribution requirements shall comply with Directive 2003/94/EC. Procedures for storage and distribution shall be validated to ensure blood and blood component quality during the entire storage period and to exclude mix-ups of blood components. All transportation and storage actions, including receipt and distribution, shall be defined by written procedures and specifications. Autologous blood and blood components as well as blood components collected and prepared for specific purposes shall be stored separately. Appropriate records of inventory and distribution shall be kept.
**Packaging**	Packaging shall maintain the integrity and storage temperature of blood or blood components during distribution and transportation.
**Return**	Return of blood and blood components into inventory for subsequent re-issue shall only be accepted when all quality requirements and procedures laid down by the blood establishment to ensure blood component integrity are fulfilled.
**External Contractors**	Tasks that are performed externally shall be defined in a specific written contract.
**Non-Conformity/Deviations**	Blood components deviating from the required and set out standards shall be released for transfusion only in exceptional circumstances and with the recorded agreement of the prescribing physician and the blood establishment physician.
**Complaints & Adverse Events**	Complaints and other information, including serious adverse reactions and serious adverse events, which may suggest that defective blood components have been issued, shall be documented, carefully investigated for causative factors of the defect and, where necessary, followed by recall and the implementation of corrective actions to prevent recurrence. Procedures shall be in place to ensure that the competent authorities are notified as appropriate of serious adverse reactions or serious adverse events in accordance with regulatory requirements.
**Recall**	There shall be personnel authorized within the blood establishment to assess the need for blood and blood component recall and to initiate and coordinate the necessary actions. An effective recall procedure shall be in place, including a description of the responsibilities and actions to be taken. This shall include notification to the competent authority. Actions shall be taken within pre-defined periods of time and shall include tracing all relevant blood components and, where applicable, shall include trace-back. The purpose of the investigation is to identify any donor who might have contributed to causing the transfusion reaction and to retrieve available blood components from that donor, as well as to notify consignees and recipients of components collected from the same donor in the event that they might have been put at risk.
**Corrective & Preventive Actions**	A system to ensure corrective and preventive actions (CAPA) on blood component non-conformity and quality problems shall be in place. Data shall be routinely analyzed to identify quality problems that may require corrective action or to identify unfavorable trends that may require preventive action. All errors and accidents shall be documented and investigated to identify system problems for correction.

**Self-Inspection/Audits**	Systems shall be in place for all parts of the operations to verify compliance with the defined standards. They shall be carried out regularly by trained and competent persons in an independent way according to approved procedures. All results shall be documented, and appropriate corrective and preventive actions shall be taken in a timely and effective manner.

Supplementary Figures

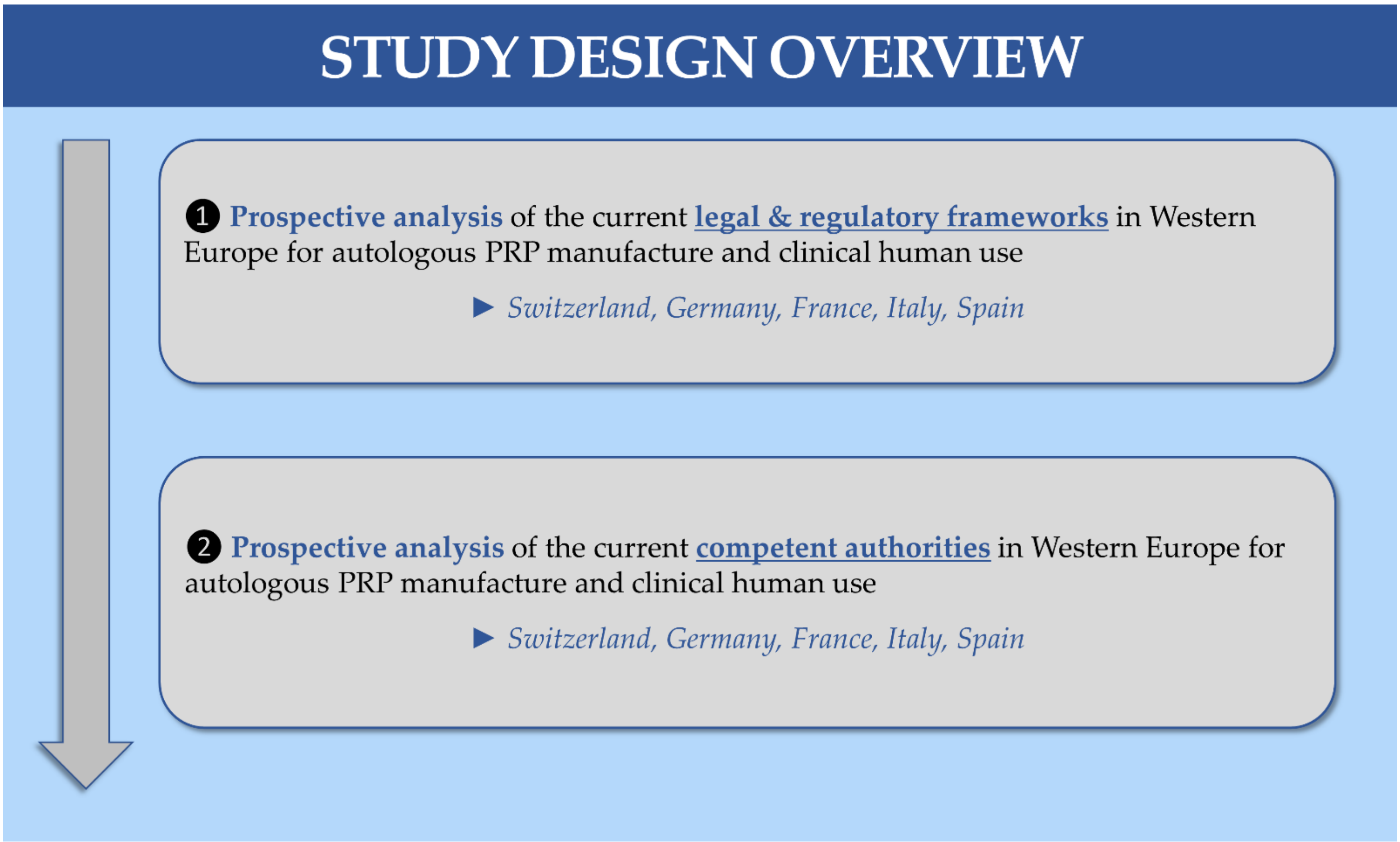

Figure S1. Schematic illustration of the design of the study. GMP, good manufacturing practices; PRP, platelet-rich plasma.

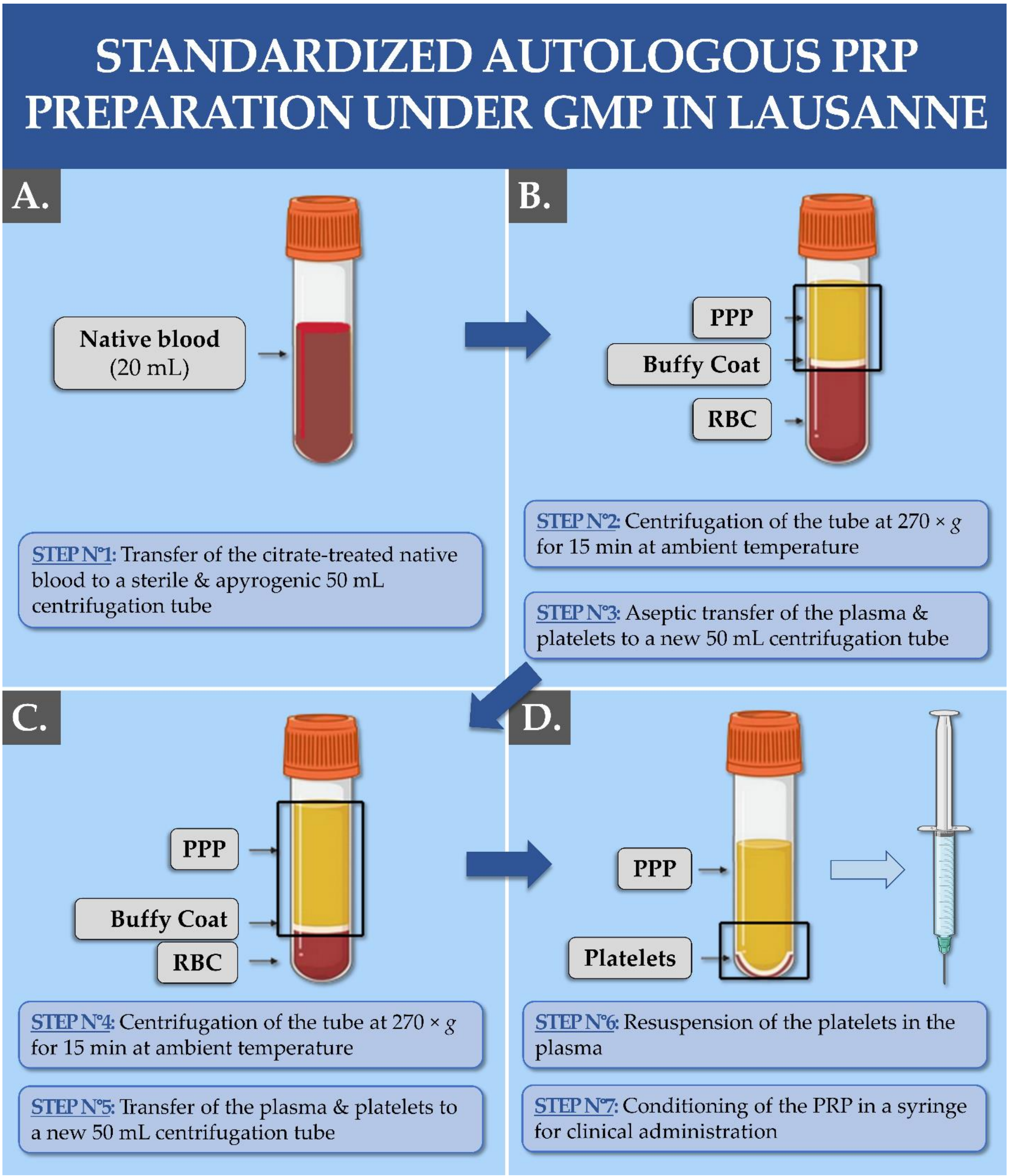

Figure S2. Schematic stepwise illustration of the standardized autologous PRP manufacturing process performed under GMP in the Lausanne University Hospital. All open-container manipulations are performed by trained and qualified personnel in a grade A GMP environment. (**A**) A small volume of whole blood (i.e., ~ 20 mL) is drawn from the patient and is prepared for centrifugation. (**B**) Following the first centrifugation step, a large amount of the RBCs is discarded. (**C**) Following the second centrifugation step, the remaining RBCs are discarded. (**D**) The obtained platelets are resuspended in the appropriate volume of autologous plasma to constitute the PRP. The PRP is then conditioned for clinical administration. Appropriate retention samples and environment samples are isolated for quality controls. GMP, good manufacturing practices; PPP, platelet-poor plasma; PRP, platelet-rich plasma; RBC, red blood cells.
